# Strategy Jams: using design thinking to charter teams with compelling purpose and sound structure

**DOI:** 10.3389/fpsyg.2026.1746557

**Published:** 2026-04-08

**Authors:** Bethany Laursen, Elizabeth LaPensee, Maureen Brudzinski, Blair Richards

**Affiliations:** Michigan Institute for Clinical and Health Research, University of Michigan, Ann Arbor, MI, United States

**Keywords:** design thinking, mixed methods, role of context, science facilitation, team charter, team chartering workshop, team science, team diagnostic survey

## Abstract

It is unclear how science facilitators can balance the amount of structure, content, and freedom they give to teams in chartering workshops. We propose that design thinking principles and activities can fill this need in team science. We piloted a team chartering workshop based on design thinking (a “Strategy Jam”) with five hybrid research/research support teams. We tracked chartering outcomes and what led to them using a mixed methods, explanatory, longitudinal study design. Data were collected using the Team Diagnostic Survey and interviews with team leaders. Immediately after the Strategy Jam, teams’ scores for their compelling purpose increased by an average of 13%. For three of the four teams measured quantitatively, their sound structure scores increased by an average of 8% while one team’s score decreased by 11%. Qualitative analysis of interviews with all five teams revealed that design thinking factors were influential, along with contextual factors and generic factors common to any participatory workshop. An unknown follow up mechanism likely contributed to further gains and losses in chartering outcomes over time. In this pilot study, design thinking was an effective and efficient way to design a participatory chartering workshop. It was effective because it helped charter team purpose and structure along with team commitment, learning, and cohesion. It was efficient because a design thinking approach fulfilled five science facilitation functions at once: (1) structure the participation process, (2) structure the workshop process, (3) facilitate generic workshop factors (e.g., aligned choosing), (4) gain traction on chartering topics for ambiguous challenges, and (5) respond to context.

## 1 Introduction

To be effective, both research teams and teams that support them need a compelling purpose and sound structure (Hackman and Wageman, 2012). Hackman and Wageman’s framework, the 6 Conditions for Team Effectiveness, has been validated with thousands of teams worldwide (Wageman et al., 2005; Hackman, 2011; Hackman and Wageman, 2012). A compelling purpose specifies the team’s goal—the clear, challenging, and consequential change they wish to make in the world. Sound structure breaks the goal into aligned, motivating tasks and establishes norms for collaborating effectively. Team strategy then emerges from purpose, structure, and other fundamental team conditions. Strategy, on this framework, is a team process of selecting appropriate ways to accomplish tasks, such as how to sequence milestones or monitor progress. Purpose and structure are so fundamental and malleable that Hackman and Wageman argue team coaches, leaders, and facilitators should focus on establishing these and other basic conditions rather than fine tuning team processes (Wageman, 2001; Hackman and Wageman, 2004, 2012; Hackman, 2012).

In science, however, grant proposals and unit mission statements rarely provide enough direction on purpose or structure to propel a team into action. Teams must often further specify and/or adapt their work designs at launch and key inflection points. Team chartering workshops can help teams clarify their purposes and plans, because team charters foster shared agreements for teamwork and taskwork (Begerowski et al., 2021). Two team chartering workshops reported in the team science literature include collaboration planning (Rolland et al., 2020, 2025) and provisional planning (Lant and Campo, 2025). However, it is not clear how science facilitators can reliably balance the amount of structure, content, and freedom they give to teams in these workshops (Ericksen and Dyer, 2004; Aaron et al., 2014; Rolland et al., 2020, 2025). There is thus an important need to understand what makes a team chartering workshop catalyze shared purpose and sound team structure. Without such principles, team chartering workshops will continue to be difficult to plan and execute.

We posit that design thinking principles can fill this need (LaPensee and Doshi, 2020). In short, design thinking is a human-centered approach to solving complex problems (Micheli et al., 2019). Design thinking can focus the content and structure of team workshops because it encourages facilitators to involve everyone in iteratively designing solutions to meet audience needs. At the University of Michigan, the Michigan Institute for Clinical & Health Research (MICHR) has developed workshops known as “Research Jams” that include design thinking principles and activities. Our Ideation and Visioning Jams reliably focus and accelerate team creativity, generating excitement for working together on a shared topic (LaPensee et al., 2021). Strategy Jams are a new workshop model aiming to convert a team’s knowledge of audience needs into actionable purposes, principles, projects, plans, performance indicators, and priorities. In other words, a Strategy Jam is a team chartering workshop based on design thinking principles and activities. While the Strategy Jam does cover team strategy (plans, performance indicators, and priorities) it emphasizes the fundamental conditions of purpose and structure (purpose, principles, and projects).

Here we present results of a pilot study with five (*N* = 5) MICHR faculty and staff teams who completed a Strategy Jam in 2024–2025. Our study aims to answer the following research questions:

1. What outcomes do teams experience from the Strategy Jam?
2. What aspects of the Strategy Jam contribute to those outcomes?
3. What is the role of design thinking principles and activities in shaping the aspects of the Strategy Jam that contribute to team chartering benefits?

By answering these questions, our study contributes to the literature on team chartering interventions (Mathieu and Rapp, 2009; Aaron et al., 2014; Brower et al., 2020; Begerowski et al., 2021) and to the practice of science facilitation (Cravens et al., 2022).

## 2 Methods

### 2.1 Participants and study setting

To fulfill its mission as a clinical and translational science hub, MICHR is organized as a multi-team system. The component teams are “programs,” each tasked with delivering research support and original research findings on issues such as team science, community engagement, and informatics. In 2024, MICHR leadership mandated that all teams revisit their strategies. Five teams agreed to complete this work through a Strategy Jam. Individual team members consented to participate in this study when they completed surveys and/or interviews.

The University of Michigan’s IRB determined this study was not regulated as human subjects research (HUM00255087). Participants are our co-workers, and in some cases, our direct reports. The following steps were taken to minimize any perceived coercion or social desirability bias: (1) no data were collected during the Strategy Jams so that workshop participants could receive the intervention without feeling compelled to participate in the research; (2) research participants were promised and given confidentiality; (3) individual responses to interviews and surveys were identifiable only to the first two authors, minimizing co-worker exposure; (4) all interviews were conducted by the first author, who is not a supervisor; and (5) research participants could decline to answer any interview or survey question.

### 2.2 Intervention

The Strategy Jam tested in this pilot study consisted of a brief pre-survey and a three-session workshop spanning 11.5 h across 5 to 6 weeks. The pre-survey requested member views of the team’s main audiences and challenges. These responses were used to populate the first activity of session one (a journey map) and were not part of this research study. Two weeks after the third session, the facilitators met with the team leaders to handoff a PDF report of the team’s Strategy Jam outcomes.

**Figure 1:**
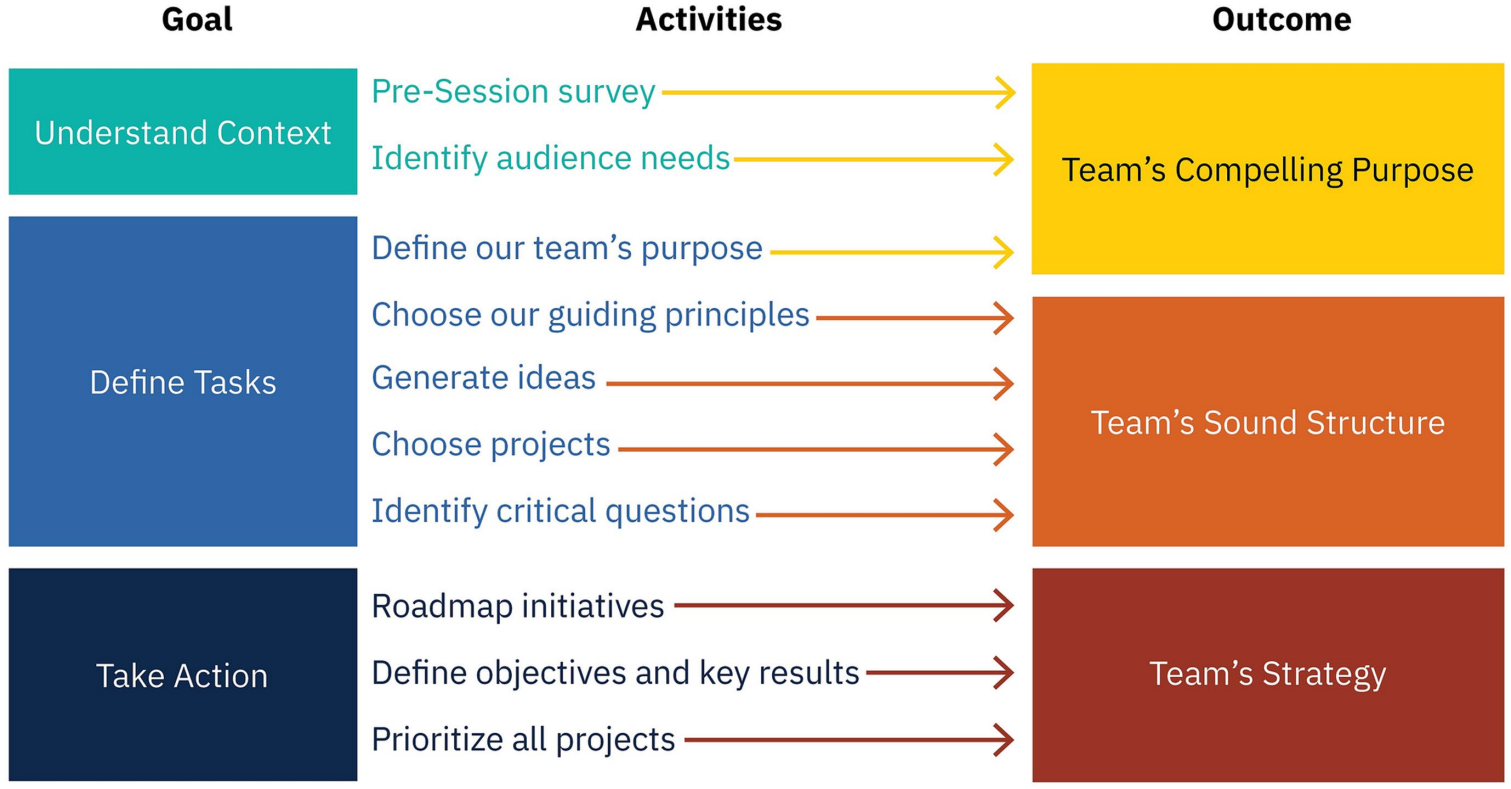
Alignment of Strategy Jam’s goals, activities, and outcomes. Divisions in this figure do not correspond to divisions between workshop sessions.

We derived the Strategy Jam from the Strategy Sprint developed by design consulting firm AJ&Smart (Courtney, 2025). Figure 1 shows how the Jam’s goals, activities, and outcomes aligned. The Strategy Jam leverages the principles that Micheli et al. (2019) note are signature to most design thinking approaches:

1. Creativity and innovation: novel, useful ideas
2. User centeredness and involvement: focusing on the needs of the team’s beneficiaries
3. Problem solving: addressing complex challenges
4. Iteration and experimentation: learning by trying
5. Interdisciplinary collaboration: integrating different perspectives
6. Ability to visualize: externalizing ideas
7. Gestalt view: holistic understanding in context
8. Abductive reasoning: imagining what might be the case
9. Tolerance of ambiguity and failure: moving forward despite uncertainty
10. Blending rationality and intuition: embracing systematic data and felt knowledge
11. Specific design tools and methods: common activities for design thinking

To implement the last principle, the Strategy Jam uses the following design activities from the list provided by Micheli et al. (2019):

- Brainstorming: We used various prompts often with a “together, alone” mechanism (Courtney, 2025) that starts with silent, individual ideation followed by group share-out and synthesis.
- Visualizations: Ideas and values are externalized on sticky notes, in templates, and in voting dots.
- Journey mapping: Mapping the phases of an audience’s journey with the team, from pain points to actions to goals.
- Prototyping: Participants drafted early-stage, simplified project ideas and timelines with high-level templates.
- Field experiments: Project prototypes were designed for experimentation in “real-world” settings.

In addition to the principles and activities specific to design thinking, the Strategy Jam uses best practices for any kind of workshop and for participatory decision making (Kaner, 2014). These generic principles include ensuring smooth logistics, tailoring the workshop to each team, and providing for equitable participation, among others detailed in the results section below.

### 2.3 Data collection

This explanatory, mixed-method study uses qualitative insights from participant interviews to interpret quantitative data collected through the Team Diagnostic Survey (Wageman et al., 2005). Four of the five teams completed the entire Team Diagnostic Survey (TDS) before the Jam (baseline, *t*_0_). We then tracked their Compelling Purpose and Sound Structure scores for up to 9 months after the Jam by repeatedly administering an abbreviated version of the TDS (Supplementary Table S1). Table 1 shows team member response rates for all time points. At times, Teams 1 and 3 did not meet the 50% response rate recommended by Wageman et al. (2005). However, we retained those scores to interpret trends across teams and over time.

**Table 1:** TDS response rates for the four teams who completed the TDS.

Team	Baseline (*t*_0_)	Before handoff (*t*_1_)	After handoff (*t*_2_)	6 months (*t*_3_)	9 months (*t*_4_)	Average
Team 1	70%	80%	45%	55%	90%	63%
Team 2	100%	100%	100%	100%	Not collected	100%
Team 3	44%	50%	38%	53%	Not collected	45%
Team 4	71%	57%	57%	Not collected	Not collected	57%

“Handoff” refers to the handoff meeting wherein the Jam facilitators delivered a PDF report of the Jam outcomes to the team leaders.

For Team 1 at *t*_1_, we used the proprietary 6 Team Conditions Pulse Check (6 Team Conditions, 2025), which uses abbreviated scales for Compelling Purpose and Sound Structure. For all other points, teams completed the full Compelling Purpose and Sound Structure scales. Although “Strategy” is a target outcome of the Strategy Jam (Figure 1), we did not track this variable over time because purpose and structure are more fundamental team charter characteristics according to the 6 Team Conditions framework (Hackman, 2012; Hackman and Wageman, 2012).

Qualitatively, for each of five teams, one or two team leaders were interviewed within 1 week of their Strategy Jam (*N* = 9; Supplementary Table S2). These semi-structured interviews focused on perceived outcomes of the Jam and what led to those outcomes (see Supplementary material). Interviews were conducted and recorded using Zoom video conferencing software. Recordings were transcribed verbatim with the machine learning module of Dovetail software (Dovetail, 2024) and reviewed by the interviewer for accuracy. When quoting interviewees below, we remove filler words such as “like” and “um.”

### 2.4 Data analysis

The quantitative analysis tracked the two target outcome variables over time: Compelling Purpose (range: 1 to 5) and Sound Structure (range: 1 to 5). Scores for these two variables were calculated as described by Wageman et al. (2005) with the following exception: to reflect recent updates to the TDS (6 Team Conditions, 2025), we excluded Team Composition from the Sound Structure scale at all time points. The outcome variables were assessed using descriptive statistics and graphical visualization to compare how the two outcomes changed over time.

The qualitative analysis aimed to uncover factors contributing to immediate Jam outcomes. The interviewer (BL) coded the interview transcripts in Dovetail. Codes were developed inductively and initially divided into two categories: (1) Jam outcomes, and (2) causes of these outcomes. Causes were further divided into three sub-categories: (a) generic workshop features, (b) features specific to design thinking, and (c) contextual factors. Codes were then compared to the list of design thinking principles from Micheli et al. (2019) and to constructs from the TDS. Inductive codes that matched any of these were renamed with the label from the relevant article; these are capitalized when reported below. See Supplementary Table S3 for final coding data and definitions. Finally, we visualized how codes were related using a process model, which showed that codes were further divided into processes and factors influencing these processes, in turn yielding Jam outcomes (Supplementary Figure S1). This model was interpreted by the first two authors as “mechanisms of impact,” where each mechanism consists of a defining process and factors that influence it, and these together produce the outcomes. In addition, given our insider knowledge of MICHR, we added factors to the process model that may have contributed to the “follow up mechanism of impact” discussed below.

## 3 Results

### 3.1 Immediately after the jam

**Figure 2:**
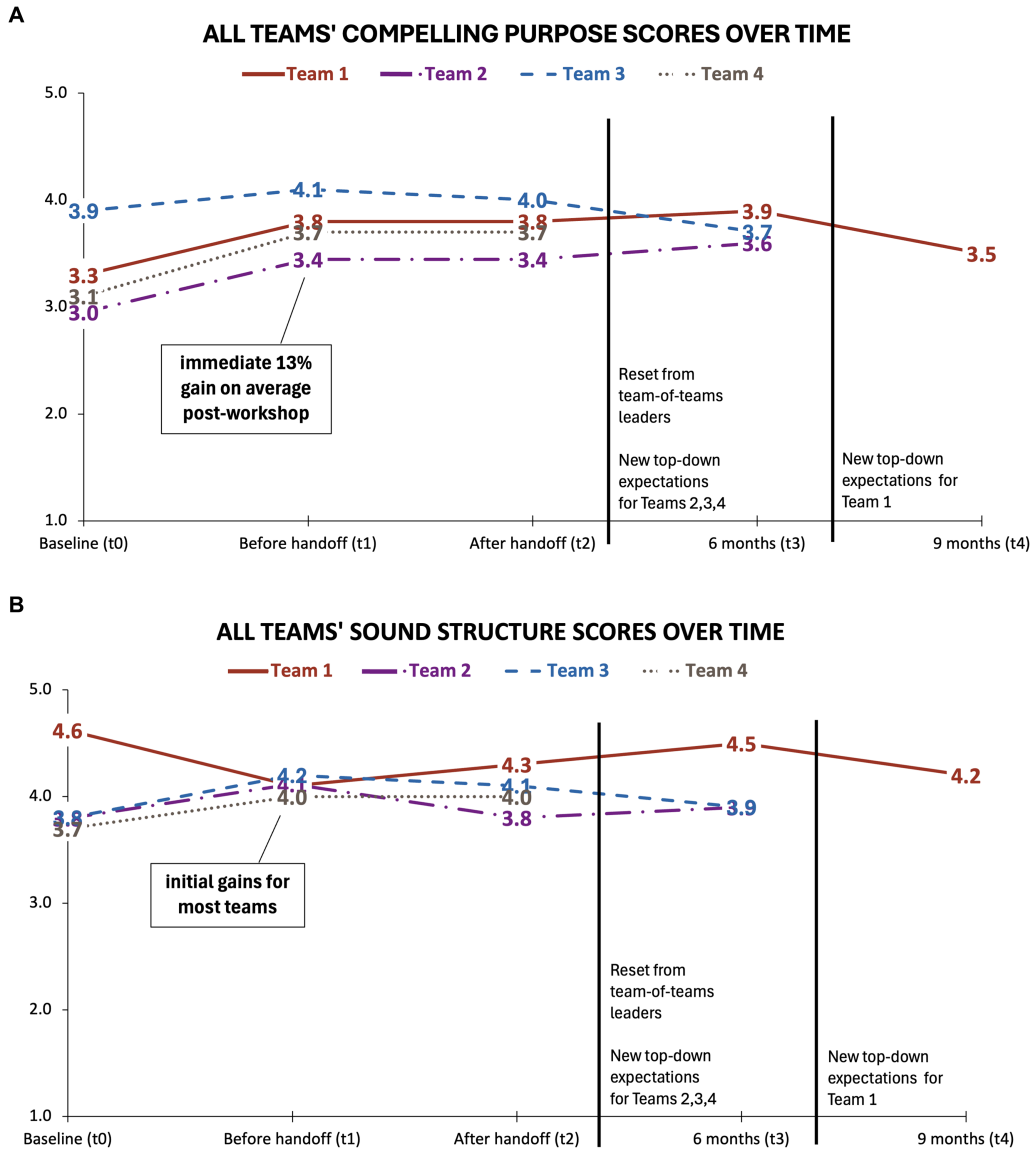
Longitudinal scores for the four teams who completed the TDS: **(A)** Compelling Purpose, and **(B)** Sound Structure.

Before the Jam, Teams 1, 2, and 4 rated their Sound Structure higher than their Compelling Purpose by an average of 0.9 out of 5. Team 3 scored nearly the same on both measures. Immediately after the Jam and before handoff (*t*_1_), the Compelling Purpose score increased by an average of 13% for all teams (Figure 2A). At the same time, Sound Structure increased an average of 8% for three of the four teams, but this score decreased by 11% for Team 1 (Figure 2B). However, this score for Team 1 may have been a measurement artifact due to using the abbreviated Pulse Check scales, in which Sound Structure was more abbreviated (2 of 11 items from the full scale) than Compelling Purpose (3 of 6 items from the full scale).

**Figure 3:**
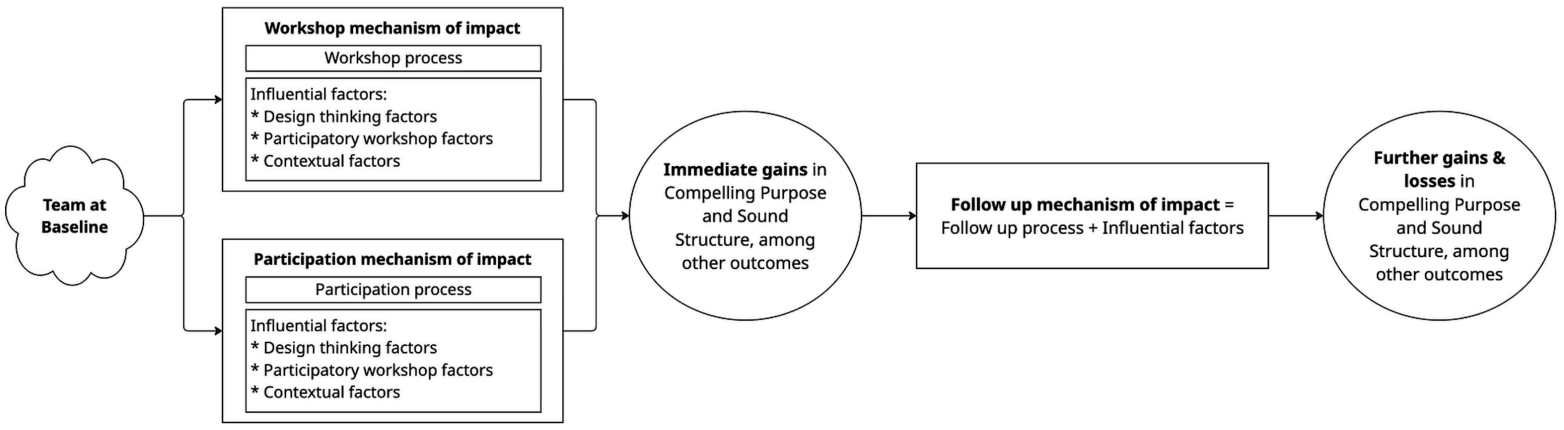
A simplified version of Supplementary Figure S1 showing the participation and workshop mechanisms, reported by interviewees, leading to chartering and other outcomes, and then to the follow up mechanism, hypothesized by the authors based on insider knowledge of MICHR events, which led to further gains and losses in team purpose and structure.

Qualitative findings corroborated the initial quantitative gains and revealed additional outcomes (Figure 3; Supplementary Figure S1). Interviewees indeed reported a Compelling Purpose as one of the most important benefits for their teams. Specifically, interviewees emphasized how clear their purpose had become. One interviewee summarized, “I think the majority of the team now understands our purpose: the things that we should be doing and who we should be serving.” Another reflected, “The purpose statement…it’s permission to say ‘No’ to things,” indicating clear direction. Surprisingly, interviewees did not comment on how Consequential or Challenging their purpose felt, even though, like Clarity, there was quantitative movement on those other two components of Compelling Purpose after the Jam (Supplementary Table S4).

Regarding Sound Structure, interviewees appreciated establishing specific projects aligned with their purpose during the Jam; we coded this as Task Design, following the TDS. An interviewee celebrated, “We’re [going to be] doing work that is advancing us to reaching a specific goal that aligns with our purpose, right? That’s strategic, not just busywork.” This felt different and exciting to interviewees, who had struggled to turn big ideas into feasible, purposeful projects. Quantitative results suggested that teams found these new tasks more Meaningful, more likely to foster Worker Autonomy, and more likely to render Visible Results (Supplementary Table S5).

In addition to Task Design, to build their Sound Structure teams also develop guiding principles during the Strategy Jam. These principles could function as Team Norms, if the team adopts them to guide member behavior. However, when asked about benefits of the Jam, interviewees did not mention these guiding principles. Still, quantitative results indicate that most teams rated their Team Norms higher after the Jam than before (Supplementary Table S5).

Interviewees were also excited about the initial work plan they generated during the Jam, which we labeled Strategy to align with the TDS (Figure 1). One interviewee went so far as to say the Strategy Jam is not worth doing if it does not culminate in Strategy: “If we had stopped at any earlier point, it would have felt good, like ‘Look at our purpose; it’s so great.’ But we would have just stalled [afterward].”

Beyond Compelling Purpose, Sound Structure, and Strategy, interviewees collectively reported three additional benefits from the Jam: (1) enthusiastic commitment; (2) learning about self, others, the team, and the organization; and (3) team cohesion. These benefits are described in section 3.1.1.

Interviews indicated two mechanisms by which immediate Jam outcomes materialized: (1) a participation mechanism, and (2) a workshop mechanism, which influences the participation mechanism (Figure 3; Supplementary Figure S1). Each mechanism consists of process(es), influencing factors (*italicized* below and defined in Supplementary Table S3), and outcomes. There were three types of influencing factors: (a) generic factors, possible with any participatory workshop, (b) design thinking factors, specific to design thinking, and (c) contextual factors, specific to MICHR’s context.

#### 3.1.1 The participation mechanism of impact

To reiterate, in this study a “mechanism of impact” consists of a defining process with its own components; factors that were influencing this process; and resulting outcomes. The participation mechanism is powered by the participation process, which is marked by best practices for any participatory meeting (Kaner, 2014; Rogelberg, 2019). Interviewees specifically noted the following participation components were important in Strategy Jams: dedicated time with colleagues, individual ideation time, having the right people in the room, and equitable participation opportunities.

There are many ways science facilitators can establish this participation process, not just design thinking methods. Still, we found that the *Ability to Visualize* was a design thinking principle that influenced participation in these Jams. One interviewee gave an example: “Voting [with sticky dots] enabled everyone to have a voice.” Interviewees appreciated *Specific Design Tools and Methods* including brainstorming, visualization, and the journey map. These design thinking principles and activities structured how individuals participated in the team conversation.

Another feature that influenced participation in our Strategy Jams was specific to our context: we had a trusted *team-of-teams leader in the room* co-facilitating the workshop. The leader’s presence signaled the importance of the Strategy Jam, motivating full engagement from the participants. One interviewee reflected, “I did not feel like I was in performance mode, but I did feel the pressure to be precise and accurate with my plans and aspirations for our program.”

The Jam’s authentic participation mechanism led to three additional outcome streams that we did not anticipate: (1) co-created ideas led to enthusiastic commitment; (2) transparency (augmented by *Ability to Visualize* and *team-of-teams leader in the room*) led to shared awareness and then to learning about self, others, the team, and the organization; and (3) the shared experience (influenced by *conducting the workshop in person*) led to stronger relationships and then to team cohesion. Because these unexpected outcomes were not the target of this study, we did not investigate them further but recommend them for future research.

#### 3.1.2 The workshop mechanism of impact

The participation mechanism was itself influenced by a second impact mechanism: the workshop mechanism. That is, we mediated participation by structuring the meeting as a workshop. Interviewees noted that the following process components, standard for any workshop, were impactful: using an external facilitator, executing smooth logistics, preparing well, designing an activity structure that flows, and tailoring the activities to the team. Note that these features distinguish a workshop from other kinds of meetings such as open discussions, status updates, or presentations (Kaner, 2014; Lipmanowicz and McCandless, 2014; Rogelberg, 2019; Courtney, 2025).

Interviewees noted many factors that influenced this generic workshop process, accounting for this unique workshop design and context. First, the Strategy Jam anchors the team’s charter in meeting the needs of their various audiences. *User-Centeredness* is a key design thinking principle that fosters clarity and commitment to solving real problems. Focusing on audiences was especially helpful in this team-of-teams context, helping teams disambiguate from each other. An interviewee reflected, “The team now understands we should not be duplicating what [Team 2] is doing, right? Our focus and our audience is [sic] much different.” This became clear to teams even though we did not share Strategy Jam outputs across teams or prompt teams to consider each other’s work.

Second, the Strategy Jam also employs a design thinking principle we call *iterative focusing* to structure the workshop. The Jam activities cycle between divergent and convergent thinking to gradually clarify an ambiguous sense of potential into an actionable team charter. Iterative focusing is a blend of design thinking principles listed by Micheli et al. (2019, p. 132), viz., “iteration and experimentation,” “tolerance of ambiguity,” and “abductive reasoning.” Iterative focusing is best captured by LaPensee and Doshi (2020, p. 2) as “bring[ing] increasing iterations of clarity to a fuzzy problem space.” Interviewees appreciated this gradual focusing because their original charge was so ambiguous and the final work plan was so clear. One interviewee observed, “We have this ‘thing’, but we do not really know what we are supposed to do or in what order we want to do them [sic]…. This [Jam] really provided some kind of structure around really focusing and prioritizing what we are doing.” Another remarked, “I’m actually amazed we got to the outcome of three things that we could do. Because I think from all the sticky notes, I’m like, Wow! How we were able to distill that down to something actionable was wonderful.”

Gradually increasing clarity was supported by gradually increasing coherence and commitment. We called this *aligned choosing*, in which teams successively cohere the various parts of their charter and commit to them. One interviewee explained,

The structured approach where you have to align what you are choosing as objectives with what you articulated earlier in the day about your purpose and challenges, that forces alignment. You don’t wanna be inconsistent, and you don’t wanna betray your articulated purpose or fail to speak to your challenges. So, there’s a momentum that’s built up or an escalation of commitment—that’s built up by the necessity of being coherent in that regard, right?

There are many ways that science facilitators can prompt aligned choosing, not just with design thinking. In the Strategy Jam, interviewees reported that the following factors facilitated this dynamic: (1) focusing the workshop on *team chartering topics* (i.e., purpose, principles, projects), (2) *Ability to Visualize* through *Specific Design Tools and Methods*, and (3) authorizing team leaders to make final decisions based on member input, which we called the *“responsive decider” decision rule*.

Focusing on *team chartering topics* is, of course, essential to any chartering workshop. In this pilot, though, the chartering focus was amplified by two contextual factors: (1) it was *good timing in the team’s lifecycle*, and (2) it was *mandated*. Many teams had just finished their first projects under a new funding source and organizational mission started in March 2022. An interviewee explained, “It was a really good point in time for us to do this as well because we are right in this stage where we are finishing up things and needing to start new things.” Another reflected, “Creating a strategy sort of makes sense at the beginning, but it actually made more sense in year two.” Regarding the mandate, an interviewee explained, “The fact that it’s mandated means that you really do have to choose. You’re not just coming up with a temporary prioritization. You’re coming up with choices. And that’s different.”

*Ability to Visualize* helped with *aligned choosing* because it created an external record of options and decisions. An interviewee explained, “You could see everybody’s ideas. You could see what people’s suggestions were for the decision. The decision usually followed those suggestions…. The flow from one thing to the next was clear.” Some interviewees believed that visualization was easier by *conducting the workshop in person*. However, this was not true for everyone. One participant had a visual impairment and we needed digital tools (e.g., Google docs) to enable their engagement with an external “visual” record. Arguably, that would have been easier with a fully remote workshop.

Interviewees explained that the *responsive decider* helped the team move forward even without 100% agreement. They observed that the *Ability to Visualize* also helped the team understand how the *responsive decider* was making decisions. An interviewee observed, “I felt really good about decisions being made in the open…. At every point, everybody’s contributions were visible. The decisions that were made were visible, and the reason why was communicated, and so it was intellectually visible.”

In sum, as with the participation mechanism, design thinking principles helped amplify the workshop mechanism, providing structures for things all participatory workshops should or could do in ways that balanced the freedom, content, and structure teams need when facing ambiguous challenges and contextual realities.

### 3.2 Longitudinal results

After *t*_1_, descriptively, scores for both Compelling Purpose and Sound Structure followed unique trajectories for each team over the remainder of the study (*t*_2_–*t*_4_) (Figure 2). Scores for Compelling Purpose were mostly sustained after the initial increase post-Jam, although Teams 1 and 3 lost ground near the end of the study while Team 2 gained ground. Results were mixed whether gains in Sound Structure were sustained or dropped off over time.

#### 3.2.1 The follow up mechanism of impact

We hypothesize but cannot confirm that longitudinal results emerge from follow up activities after the Jam, which we term the “follow up mechanism of impact.” The follow up process was not coordinated but included, at minimum, a handoff meeting 2 weeks after the Strategy Jam, further interventions from team-of-teams leaders, and team and individual engagement with elements of the team’s charter. This process was probably influenced by the following contextual factors.

Although we did not collect qualitative data after *t*_1_, our insider knowledge of MICHR reveals two back-to-back events that formed an inflection point for all teams. As shown in Figure 2, this inflection occurred between *t*_3_ and *t*_4_ for Team 1 and between *t*_2_ and *t*_3_ for the other teams. First, there was a shift in organizational strategy from leaders of the multi-team system. They directed all teams to revisit outputs from their Strategy Jams and dig deeper into audience needs in line with a narrower organizational mission. Second, soon thereafter, stakeholder expectations evolved from the top down, further influencing team purposes and plans. Figure 2 indicates that each team responded differently to this inflection point.

## 4 Discussion

### 4.1 Role of design thinking

In summary, structuring this team chartering meeting as a workshop mediated participation and resulted in a more Compelling Purpose especially by increasing Clarity, a more Sound Structure especially through aligned Task Design, and other benefits. Several features of this particular workshop shaped the outcomes. Some of these features, such as *aligned choosing*, can be accomplished with a variety of facilitation approaches. Other features, such as *User-Centeredness*, are unique to design thinking. And still other features, such as *timing in the team lifecycle*, will vary in each context. Workshop impacts changed over time, likely with the follow up mechanism.

Our results are consistent with Ericksen and Dyer (2004), who found that team launch meetings were more effective if they were participatory rather than command-and-control. We found that design thinking is one way to structure such a meeting when the approach is embedded in a robust workshop process. Specifically, the following design thinking principles were influential in Strategy Jams:

- User-centeredness
- Ability to visualize
- Iterative focusing (a blend of iteration and experimentation, tolerance for ambiguity and failure, and abductive reasoning)
- Specific design tools and methods (viz., brainstorming, visualizations, journey mapping, prototyping, field experiments)

In this pilot study, design thinking was an effective and efficient way to design a participatory chartering workshop in team science. It was effective because it helped charter team purpose and structure along with team commitment, learning, and cohesion. It was efficient because a design thinking approach fulfilled five science facilitation functions at once: (1) structure the participation process, (2) structure the workshop process, (3) facilitate generic workshop factors (e.g., aligned choosing), (4) gain traction on chartering topics for ambiguous challenges, and (5) respond to context.

### 4.2 Study limitations

As a pilot, this study has several limitations that limit reliability and generalizability. First, participating teams were hybrid research/research support teams and thus may not have responded to the Strategy Jam the way dedicated research teams would. For example, because research teams might tend to view their work as filling knowledge gaps rather than meeting other people’s needs, the idea of identifying audience needs might be a source of confusion rather than a step towards clarity. Second, we did not collect data on the follow up mechanism of impact, so we cannot be sure what explains the long-term outcomes for each term. Third, the quantitative trends are uncertain, because teams participated in the study for different lengths of time, a slightly different Pulse Check survey was used for one data point, Teams 1 and 3 had different members responding at different time points, and survey response rates were less than ideal for some time points. Fourth, while the TDS has been validated with thousands of teams of many kinds, reliability and validity are unknown for this specific kind of team. Fifth, there are no published norms for the TDS that might indicate typical baselines for this kind of team or to what extent scores typically change after an intervention. We do not know if 8–13% is a small, modest, or large gain for Compelling Purpose and Sound Structure. Lastly, this pilot does not address how to choose appropriate chartering topics for science teams, although our findings indicate that team purpose, task design, and strategy were appreciated.

### 4.3 Future directions and conclusion

This pilot study indicates promising directions for the practice and study of team science:

- Design thinking is one approach to science facilitation that can charter team purpose and structure when embedded in a sound workshop process.
- Science facilitators can leverage contextual factors to amplify the impact of chartering workshops. Examples include timing the workshop before/after major phases of work (Gersick, 1988; Luvison and Michel, 2025), responding to organizational mandates, and including a trusted organizational leader in the workshop.
- Comparative case studies or quasi-experimental designs are needed to discern if the design thinking factors found to be influential in this pilot yield differential advantages over other approaches to workshop facilitation, such as agile methodology (Morrison et al., 2019), Technology of Participation (Wayne, 2017), or Liberating Structures (Lipmanowicz and McCandless, 2014).
- Comparative research is also needed to determine how different contextual factors influence chartering outcomes immediately and over time.
- Further research is required to understand how the mechanisms of impact might differ for research teams versus these hybrid teams, and for different kinds of research teams (e.g., long-term labs vs. three-year projects).
- Questions remain on how to choose chartering topics for science teams. Future studies could track longitudinal outcomes from workshops focusing on different chartering topics (cf. Kanaga and Prestridge, 2002; Hackman et al., 2009; Rapp, 2025; Rolland et al., 2025). These would need to account for differences in team types and contexts.
- When studying team chartering interventions, the TDS is a promising instrument. However, TDS reliability and validity need to be determined for these populations. Moreover, there is a need for robust longitudinal data, especially qualitative data, to understand quantitative trends.

## Data Availability

The original contributions presented in the study are included in the article/Supplementary material, further inquiries can be directed to the corresponding author.
