# Supplementary material for: Strategy Jams: using design thinking to charter teams with compelling purpose and sound structure
Source: Front Psychol. 2026 Apr 8;17:1746557. doi: 10.3389/fpsyg.2026.1746557 (PMC13099884; doi:10.3389/fpsyg.2026.1746557)

***Supplementary Material***

# 1 Supplementary Information: Semi-structured Interview Protocol

1. What is your role with your program?
2. How long have you been with MICHR?
3. What are your initial reactions to the Strategy Jam we recently completed with your program? (“How did it go?”)
4. What did you personally get out of the Strategy Jam?
5. What did your team get out of the Strategy Jam? Probes: Learning? Behavior changes? Long-term results? (Anticipated)
   - Which of these do you think is the most important benefit?
   - What factors do you think were crucial to reaching these benefits? For example, anything about the activities, or who was there, or the place where we met?
6. What was negative about the Strategy Jam?
7. What are the next steps for your team after the Strategy Jam?
   - What support does your team need, if any, to take these next steps?
8. What was helpful in preparing for the Strategy Jam? (either facilitators’ or participant prep)
9. What could be improved in preparing for the Strategy Jam? (either facilitators’ or participant prep)
10. Overall, how valuable did you find the Strategy Jam? (1-10, where 10 is Extremely Valuable)
    - If 9 or 10, "What's the main reason for your score?"
    - If 0 thru 8, "What could have improved your score?"
11. If you could summarize your team's Strategy Jam in one word, what would be? Why?

# 2 Supplementary Tables and Figures

## **2.1** **Supplementary Tables (continued on next page)**

Table S1: Timing of administrations of the TDS. “NA” = Not applicable, because data were not collected.

| **Team** | **Strategy Jam session 3 date (YYYY-MM-DD)** | **Baseline (t_0_)** | **Before handoff report (t_1_)** | **After handoff report (t_2_)** | **6 months (t_3_)** | **9 months (t_4_)** |
| --- | --- | --- | --- | --- | --- | --- |
| Team 1 | 2024-08-07 | pre-Jam | 2 weeks later | 22 weeks | 26 weeks | 44 weeks |
| Team 2 | 2025-01-08 | pre-Jam | 1 week later | 6 weeks | 22 weeks | NA |
| Team 3 | 2024-12-17 | pre-Jam | 1 week later | 8 weeks | 25 weeks | NA |
| Team 4 | 2024-12-23 | pre-Jam | 3 weeks later | 8 weeks | NA | NA |
| Team 5 | 2024-07-23 | NA | NA | NA | NA | NA |

Table S2: Interviewee profiles.

| **Interviewee ID #** | **Team** | **Position Title** | **MICHR Tenure (# years)** |
| --- | --- | --- | --- |
| 1 | Team 1 | Program Director | 16 |
| 2 | Team 1 | Program Manager | 1 |
| 3 | Team 2 | Program Coordinator | 9 |
| 4 | Team 2 | Program Director | 4 |
| 5 | Team 3 | Program Director | 16 |
| 6 | Team 3 | Faculty Lead | 2 |
| 7 | Team 4 | Program Director | 16 |
| 8 | Team 5 | Program Manager | 1 |
| 9 | Team 5 | Program Director | 8 |

| **Code group** | **Code title** | **Code description** | **Number of interviewees** | **Number of segments** | **Example segment** |
| --- | --- | --- | --- | --- | --- |
| Contextual Factors | Good Timing | Teams were reflecting on their progress and strategy at a pivotal point in their development. | 3 | 6 | "It was a really good point in time for us to do this as well, like, because we are right in this in the stage where we're finishing up things and needing to start new things" (Team 1) |
| Contextual Factors | Organizational Direction | The overall direction of the larger organization shaped understanding of team purpose. | 4 | 3 | "We can remember that, right, like MICHR is doing CTS, right? We are doing CTS and we need to be thinking about ways in which our program can do that in addition to serving our, our community, our researcher, research team, community, and because I think that's gonna benefit MICHR, right?" (Team 4) |
| Contextual Factors | Team-of-Teams Leader in the Room | Having a team-of-teams representative in the room amplified impact by increasing awareness of organizational resources and team commitment to choices. | 8 | 2 | "I think there was a lot of cultural learning that happened by having [our director] there and you there." (Team 1) |
| Contextual Factors | Organizational Mandate | The top-down mandate from leadership to revisit team strategy provides a higher level of commitment and accountability, as opposed to a more informal exercise. | 6 | 1 | "The fact that it's mandated means that you really do have to choose. You're not just coming up with a temporary prioritization, you're coming up with choices and that's different." (Team 5) |
| Design Specific Features | Ability to Visualize | Making ideas visual allowed participants to see how their individual approaches and thought processes aligned and complemented each other, fostering a shared understanding as well as accountability and buy-in. | 8 | 19 | "It was helpful for me to have [my counterpart's] ideas written down, like especially when it came to her interpretation of our challenges, our purpose, our objectives. Seeing those things articulated in her own words, in a structured format was a huge benefit." (Team 5) |
| Design Specific Features | Iterative Focusing | The workshop had a focusing effect that allowed participants to distill their big ideas into actionable plans in a logical progression. | 7 | 11 | "I do think that going, like the two days all together, like going all the way from like what's our purpose? Why are we here? Like, what do we do, but why--why do we exist as a team? All the way to like in months 1 through 3 we're gonna be like doing these things and like obviously everything that's in between." (Team 2) |
| Design Specific Features | User Centeredness | Focusing on understanding user needs and tailoring the team's approach to different audience segments helped to clarify team purpose and avoid overlap with other teams. | 4 | 9 | "The team definitely, I think the majority of the team now understand our purpose. The things that we should be doing and who we should be serving." (Team 4) |
| Design Specific Features | Specific Design Tools and Methods | Participants appreciated brainstorming, visualization, and journey mapping. | 5 | 6 | "We added based on the input of the group and our community partner, another audience [to the journey map]….I think that was really key again about voice and inclusion." (Team 3) |
| Generic Workshop Features | Aligned Choosing | Participants value choosing tasks, projects, and priorities that align with a clear purpose and mission. | 7 | 22 | "This aligns up with, you know, our priorities, and these are things that we really need to do and we need to make this happen." (Team 5) |
| Generic Workshop Features | Responsive Decider | A Responsive Decider considers everyone’s input and then makes a decision for the good of the group. | 3 | 8 | "I love the fact that you all use the decision making process so that decisions could be made in real time and we weren't like kind of waiting for decisions to be made later. I think it was really helpful for decisions to be made in that moment." (Team 4) |
| Generic Workshop Features | Individual Differences | Individuals experienced the Jam differently. | 3 | 4 | "I really, personally, wanna follow through. Maybe that varies across people who participate." (Team 5) |
| Outcomes | Compelling Purpose | The Strategy Jam provided a clear sense of direction and permission to say no to requests that do not align with the defined purpose. | 7 | 30 | "It's really understanding if we're going to do something right, we have to know why we're doing it. And we have to do it together. And I feel like now we've answered that why and that whole question and now we can move in the right direction." (Team 4) |
| Outcomes | Task Design | Specific tasks (projects) were outlined as experiments for achieving the team's overall purpose. | 9 | 27 | "limited number of complementary objectives." (Team 5) |
| Outcomes | Positive Reaction | Participants generally enjoyed the event and found it exciting, impactful, and engaging. | 7 | 20 | "I'm really excited that our team went through it and I'm glad that I'll be able to be a cheerleader for other people, other people going through it." (Team 4) |
| Outcomes | Knowledge Gain | Participants learned about themselves, each other, their team, and the organization. | 9 | 19 | "I imagine for them there was a lot of learning that was accomplished" (Team 3) |
| Outcomes | Overall Value | The Strategy Jam was a valuable and worthwhile experience. | 9 | 19 | "I do think it was very valuable to get us on the right track." (Team 1) |
| Outcomes | Group Cohesion/Commitment | The Strategy Jam fostered team cohesion and commitment to project outcomes. | 7 | 19 | "team building and community and hearing voice, respect. Facilitating cohesion." (Team 3) |
| Outcomes | Strategy | Teams appreciated having tangible outcomes and a timeline for implementing the discussed ideas, rather than just broad mission statements. | 7 | 14 | "I think we came out with a lot of ideas and plans for our next steps." (Team 4) |
| Outcomes | Shared Awareness | The Strategy Jam helped create awareness and understanding of the team's purpose and initiatives, especially for newer or more peripheral members. | 6 | 14 | "I think it was just like we gave voice to all the initiatives, you know, that there was information exchange and sharing across all the initiatives." (Team 3) |
| Outcomes | Behaviors | Specific strategies, such as "can we" questions, were identified as potentially beneficial to continue using in future meetings and problem-solving sessions. | 7 | 10 | "I do think that we as leaders would have to maybe provide that scaffolding and guidance and that explicit instruction of let's approach it." (Team 3) |
| Outcomes | Needed Follow Up | Follow-up and ongoing support are important to ensure the successful implementation of new initiatives and plans. | 6 | 9 | "Maybe just, you know, like friendly reminders at different time points along the way, like, how, how is your road map going?" (Team 2) |
| Outcomes | Unintended Positive | The Strategy Jam went beyond its intended objectives, leading to serendipitous interactions, personal growth, and a strengthened sense of community among the participants. | 6 | 8 | "I always love like the little personal like inside jokes that come out of like moments like that" (Team 2) |
| Outcomes | Overwhelm | Participants feel overwhelmed by the amount of work and new projects that have been laid out for them. | 4 | 6 | "When you produce a bunch of stuff, sometimes it just takes it out of you." (Team 5) |
| Outcomes | Leader Validation | Team leaders felt a sense of validation and gratification from the insights and initiatives that were generated and agreed upon by the team. | 3 | 6 | "It kind of validated what I was feeling and some of the efforts that [my counterpart] and I have been engaged in to try to promote community and belonging and inclusion." (Team 3) |
| Participation Process | Participation | Everyone had a voice, contributed ideas, and was involved in the decision-making process. | 9 | 39 | "It was really great to, I have the time and that space to talk with our team and here are like other people's priorities." (Team 2) |
| Participation Process | Who's in the Room | The optimal group size and composition seems to be a core team of key decision-makers, with opportunities to engage a broader set of stakeholders at different stages. | 8 | 30 | "I think it definitely has to do with who was in the room." (Team 2) |
| Participation Process | Dedicated Time | The teams appreciated the dedicated time they had to come together and reflect on their work, discuss the program's direction, and generate ideas collaboratively. | 7 | 18 | "So giving that time and space for you to have those more inclusive conversations" (Team 1) |
| Participation Process | Horizontal Distribution of Responsibility | The Strategy Jam process encourages a collaborative and participatory approach to decision-making, where everyone is involved in the process and feels a sense of ownership over the collective decisions. | 4 | 10 | "I think it was just the the brainstorming process. I think it was just having all the minds kind of working cohesively." (Team 1) |
| Participation Process | Individual Ideation Time | The use of individual ideation with sticky notes prior to group discussion helped participants with different ideation preferences to contribute more equally. | 2 | 2 | "I think it's always good to give people, even if it's brief, to give people time for self reflection and to be able to articulate briefly what they're thinking before they're asked to respond or comment on, you know, what others are thinking." (Team 3) |
| Workshop Process | Preparation | The pre-work, including surveys and assessments, was generally viewed as helpful in preparing participants for the Strategy Jam. | 9 | 22 | "I haven't thought about the role that pre-survey played. It didn't hurt, it was not difficult to fill out. Didn't take a lot of time. I know it helped, the facilitation on the facilitation side of things." (Team 5) |
| Workshop Process | Logistics Pains | Participants faced several logistical challenges in organizing and attending the meetings. | 8 | 15 | "That five hour time block is a real barrier." (Team 5) |
| Workshop Process | External Facilitator | Expert facilitation enabled participants to fully engage in the strategy discussions without having to worry about the logistics and flow of the sessions. | 7 | 12 | "So to be able to have a session that was facilitated that kept us on track, that You know, we had clear milestones that we had to hit. This, it's just really nice." (Team 2) |
| Workshop Process | This Overall Structure | Participants generally found the structured activities and sessions helpful, though some initial confusion was experienced, particularly for those who did not attend the first day. | 6 | 12 | "I really do love how it everything builds on each other. It makes it really easy." (Team 2) |
| Workshop Process | The Room | Features of the room can provide or hinder a welcoming, collaborative atmosphere. | 1 | 3 | "I did like the shape of the room, the first one. I know this sounds crazy, but like I think that was a factor." (Team 3) |
| Workshop Process | Having a Structure | Having a structured approach was helpful in generating insights. | 2 | 3 | "Like, being able to, like, actually do that in a structured way, I think was really helpful." (Team 1) |
| Workshop Process | Tailored to the Team | Customizing activities and content for the specific team is important. | 2 | 2 | "It was, you know, specifically for our program and I, that I have never done that to that level." (Team 1) |

Table S4: TDS scores for Compelling Purpose and all subscales

| **Timepoint** | **Team** | **Compelling Purpose** | **Challenge** | **Clarity** | **Consequence** |
| --- | --- | --- | --- | --- | --- |
| Baseline (t_0_) | 1 | 3.3 | 2.3 | 3.3 | 4.3 |
| Before handoff (t_1_) | 1 | 3.8 | 3.3 | 3.7 | 4.3 |
| After handoff (t_2_) | 1 | 3.8 | 2.8 | 4.0 | 4.5 |
| 6 months (t_3_) | 1 | 3.9 | 2.9 | 4.2 | 4.8 |
| 9 months (t_4_) | 1 | 3.5 | 3.2 | 3.4 | 3.2 |
| Baseline (t_0_) | 2 | 3.0 | 2.8 | 2.6 | 4.0 |
| Before handoff (t_1_) | 2 | 3.4 | 2.3 | 3.8 | 4.5 |
| After handoff (t_2_) | 2 | 3.4 | 2.3 | 3.8 | 4.5 |
| 6 months (t_3_) | 2 | 3.6 | 2.6 | 3.5 | 4.3 |
| Baseline (t_0_) | 3 | 3.9 | 3.6 | 3.6 | 4.6 |
| Before handoff (t_1_) | 3 | 4.1 | 3.6 | 3.9 | 4.9 |
| After handoff (t_2_) | 3 | 4.0 | 3.4 | 3.8 | 4.8 |
| 6 months (t_3_) | 3 | 3.7 | 3.9 | 2.8 | 4.5 |
| Baseline (t_0_) | 4 | 3.1 | 3.1 | 2.0 | 4.2 |
| Before handoff (t_1_) | 4 | 3.7 | 3.0 | 3.4 | 4.6 |
| After handoff (t_2_) | 4 | 3.7 | 3.1 | 3.3 | 4.8 |

Table S5: TDS scores for Sound Structure and all subscales

| **Timepoint** | **Team** | **Sound Structure** | **Norms** | **Task Design** | **Worker Autonomy** | **Visible Results** | **Meaningful Tasks** |
| --- | --- | --- | --- | --- | --- | --- | --- |
| Baseline (t_0_) | 1 | 4.6 | 4.7 | 4.6 | 4.5 | 4.5 | 4.7 |
| Before handoff (t_1_) | 1 | 4.1 | 4.1 | 4.1 | Not collected | | |
| After handoff (t_2_) | 1 | 4.3 | 4.7 | 4.2 | 4.6 | 4.3 | 4.0 |
| 6 months (t_3_) | 1 | 4.5 | 4.8 | 4.4 | 4.3 | 4.5 | 4.4 |
| 9 months (t_4_) | 1 | 4.2 | 4.3 | 4.2 | 4.0 | 4.1 | 4.5 |
| Baseline (t_0_) | 2 | 3.8 | 3.5 | 4.0 | 4.3 | 3.5 | 4.3 |
| Before handoff (t_1_) | 2 | 4.1 | 3.8 | 4.2 | 4.4 | 3.6 | 4.8 |
| After handoff (t_2_) | 2 | 3.8 | 3.7 | 3.9 | 4.3 | 3.0 | 4.6 |
| 6 months (t_3_) | 2 | 3.9 | 3.9 | 3.9 | 3.8 | 3.8 | 4.1 |
| Baseline (t_0_) | 3 | 3.8 | 3.6 | 4.0 | 3.5 | 4.0 | 4.3 |
| Before handoff (t_1_) | 3 | 4.2 | 4.0 | 4.3 | 4.1 | 4.2 | 4.5 |
| After handoff (t_2_) | 3 | 4.1 | 3.3 | 4.4 | 4.3 | 4.0 | 4.7 |
| 6 months (t_3_) | 3 | 3.9 | 3.8 | 4.0 | 3.9 | 3.7 | 4.4 |
| Baseline (t_0_) | 4 | 3.7 | 3.2 | 3.9 | 4.0 | 3.7 | 4.2 |
| Before handoff (t_1_) | 4 | 4.0 | 3.9 | 4.1 | 3.7 | 4.3 | 4.1 |
| After handoff (t_2_) | 4 | 4.0 | 3.7 | 4.0 | 3.9 | 3.9 | 4.3 |


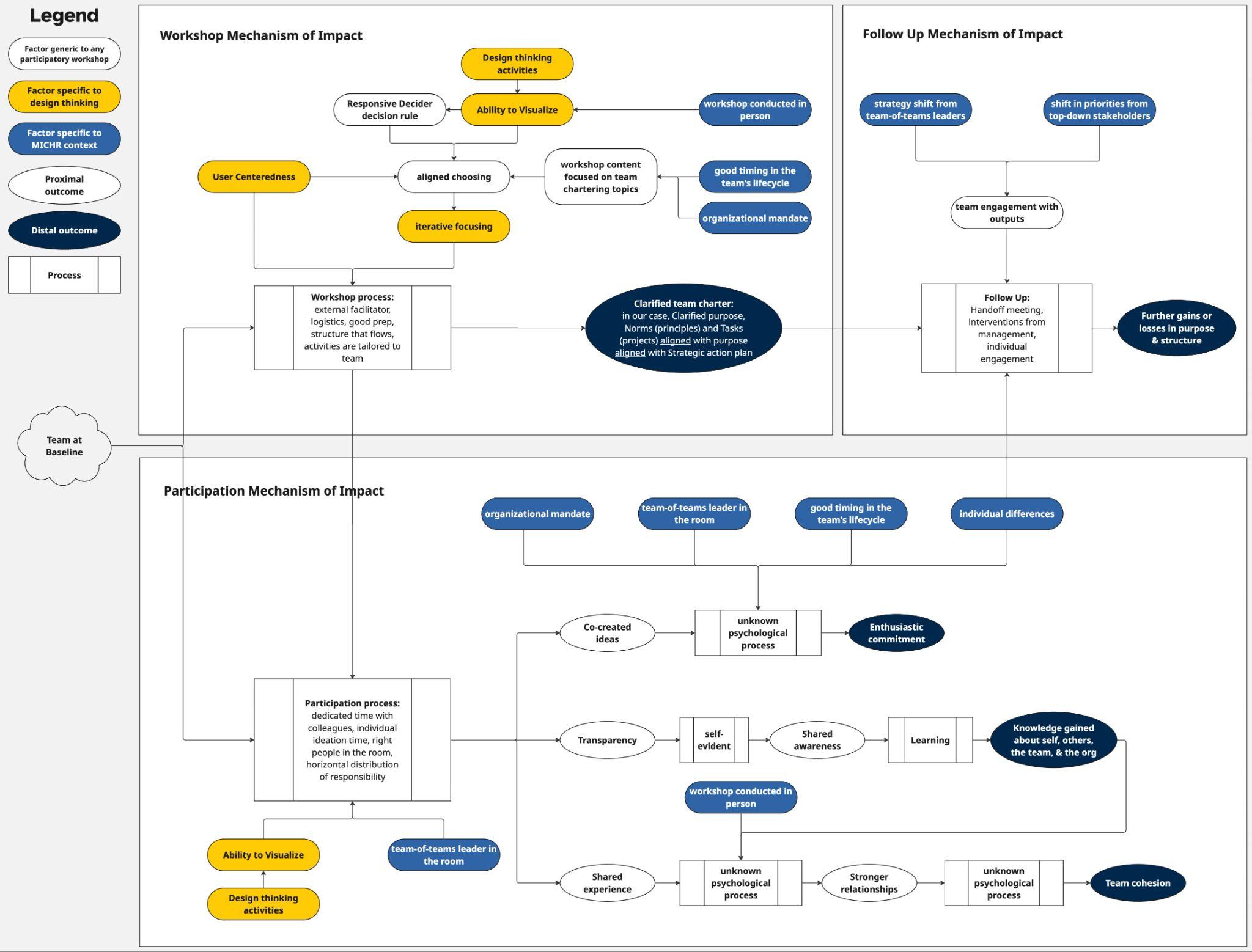

Supplement: Supplementary file 1 [file Table_1.docx]
